# A Deep Learning Model for Cell Growth Inhibition IC50 Prediction and Its Application for Gastric Cancer Patients

**DOI:** 10.3390/ijms20246276

**Published:** 2019-12-12

**Authors:** Minjae Joo, Aron Park, Kyungdoc Kim, Won-Joon Son, Hyo Sug Lee, GyuTae Lim, Jinhyuk Lee, Dae Ho Lee, Jungsuk An, Jung Ho Kim, TaeJin Ahn, Seungyoon Nam

**Affiliations:** 1 Department of Health Sciences and Technology, Gachon Advanced Institute for Health Sciences and Technology, Gachon University, Incheon 21999, Korea; chan7844@naver.com (M.J.); parkar13@gmail.com (A.P.); drhormone@naver.com (D.H.L.); 2 VUNO Inc., Seoul 06536, Korea; ryanistic@gmail.com; 3 Samsung Advanced Institute of Technology, Suwon, Gyeonggi-do 16678, Korea; wonjoon.son@samsung.com (W.-J.S.); hyosug@samsung.com (H.S.L.); 4 Korean Bioinformation Center (KOBIC), Korea Research Institute of Bioscience and Biotechnology (KRIBB), Daejeon 34141, Korea; gyutae@kribb.re.kr (G.L.); jinhyuk@kribb.re.kr (J.L.); 5 Department of Bioinformatics, University of Sciences and Technology, Daejeon 34113, Korea; 6 Department of Internal Medicine, Gachon University Gil Medical Center, Gachon University School of Medicine, Incheon 21565, Korea; junghokimm@gilhospital.com; 7 Department of Pathology, Gachon University Gil Medical Center, Gachon University School of Medicine, Incheon 21565, Korea; anbox@naver.com; 8 Department of Life Sciences, Handong Global University, Pohang 37554, Korea; taejin.ahn@handong.edu; 9 Department of Genome Medicine and Science, College of Medicine, Gachon University, Incheon 21565, Korea; 10 Department of Life Sciences, Gachon University, Seongnam 13120, Korea; 11 Gachon Institute of Genome Medicine and Science, Gachon University Gil Medical Center, Incheon 21565, Korea

**Keywords:** artificial intelligence, drug responsiveness prediction, drug discovery

## Abstract

Heterogeneity in intratumoral cancers leads to discrepancies in drug responsiveness, due to diverse genomics profiles. Thus, prediction of drug responsiveness is critical in precision medicine. So far, in drug responsiveness prediction, drugs’ molecular “fingerprints”, along with mutation statuses, have not been considered. Here, we constructed a 1-dimensional convolution neural network model, DeepIC50, to predict three drug responsiveness classes, based on 27,756 features including mutation statuses and various drug molecular fingerprints. As a result, DeepIC50 showed better cell viability IC50 prediction accuracy in pan-cancer cell lines over two independent cancer cell line datasets. Gastric cancer (GC) is not only one of the lethal cancer types in East Asia, but also a heterogeneous cancer type. Currently approved targeted therapies in GC are only trastuzumab and ramucirumab. Responsive GC patients for the drugs are limited, and more drugs should be developed in GC. Due to the importance of GC, we applied DeepIC50 to a real GC patient dataset. Drug responsiveness prediction in the patient dataset by DeepIC50, when compared to the other models, were comparable to responsiveness observed in GC cell lines. DeepIC50 could possibly accurately predict drug responsiveness, to new compounds, in diverse cancer cell lines, in the drug discovery process.

## 1. Introduction

Drug responsiveness prediction is ultimately related to precision medicine (i.e., individualized therapy), to improve cancer patient treatment benefits [1,2,3]. For example, gastric cancer (GC) has been known as one of the heterogeneous cancer types [4,5]. Even though trastuzumab was approved by the U.S. Food and Drug Administration (FDA), in combination with chemotherapy, for HER2^+^ GC patients [6], only 2–7% of diffuse type GC patients were known to be HER2^+^ [7]. So, it indicates that trastuzumab responsiveness is different even in the same GC patients [5,6]. In general, one challenge to such prediction is differential drug responsiveness, even within heterogeneous cell subpopulations in the same tumor [3]. It has now been well established, in the field of cancer omics, that these phenotypic differences align with heterogeneous patient genomic profiles, using datasets such as The Cancer Genome Atlas (TCGA) [8]. Despite the availability of such data, drug response prediction remains challenging, and it is likely unfeasible to observe drug responsiveness while treating large patient populations due to the cost of follow-up [9]. Instead, treatment-induced phenotypes, by various antineoplastics, on diverse cancer cell lines, could yield drug treatment data (e.g., cell viability, drug half-maximal growth inhibition (IC50)). Such phenotypes are associated with cell line genomics and transcriptomics information, recognized tools for predicting drug-responsiveness [10,11,12,13,14,15]. Currently, drug response prediction has been limited to similarities/differences of gene expression profiles, between cells. When gene expression (equivalently, transcriptomics) profiles of two drugs are similar to each other, it is assumed that drug response to the specific drugs is also similar [14,15]. However, unlike transcriptomics information, genomics information (e.g., mutations, indels, etc.) has not been explored for successful drug-responsiveness prediction [16,17].

To address this drug response prediction conundrum, machine learning (ML) [3,18,19,20], and network biology [9] approaches have been used. However, areas under the receiver operating characteristic (AUROC or AUC) curves, based on machine learning methods for predicting transcriptome-based drug responsiveness, have only been moderate (>0.6) [9,21]. In these methods, drug features, including molecular descriptors and fingerprints [22], i.e., abstract 1-dimensional, 2-dimensional, and 3-dimensional structures of drugs, well recognized in in the field of computational chemistry, have not been considered.

To date, drug response prediction, by integrating cell line genomics profiles (somatic mutations or transcriptomics), and drug features (drug molecular descriptors/fingerprints), has not been attempted, using ML algorithms. Recently however, a deep neural network, cancer drug response profile scan (CDRScan) was created, using cell line genomics profiles and drug molecular properties for input [17]. Even so, this approach considered binary classification accuracy (i.e., either low or high drug responsiveness) for drug responsiveness, and binary classification is not ideal.

GC is the fifth most common cancer with 783,000 estimated deaths [23,24]. Gastric adenocarcinomas have been involved in *Helicobacter pylori* and Epstein–Barr virus [25]. In case of hereditary diffuse GC, *CDH1* germline mutations have been recognized [25]. In early GC, surgical removal is the effective treatment. Due to few symptoms in early GC, most GC patients are found at advanced stages. As GC histology classification, Lauren classification and World Health Organization (WHO) classification have been widely used [26]. According to the GC progression model for Lauren intestinal and diffuse subtypes [27], both subtypes had common sequential molecular events including *TP53* mutation and cyclin E over-expression/amplification [26]. The two Lauren subtypes (diffuse, intestinal) also had their specific genetic events including *KRAS* mutations and *MET* amplification [26]. In general, Lauren intestinal subtype progressed from normal epithelia to early well-differentiated cancer through the sequence of *Helicobacter pylori* infection, cyclin E over-expression, and mutations of *APC*, *TP53*, and *KRAS*. Then, the early well-differentiated cancer suffered from HER2 amplification, leading to advanced cancer and metastasis [26]. For Lauren diffuse subtype, normal epithelia suffered from histone deacetylation, *TP53* mutation, and cycle E overexpression, leading to poorly differentiated early cancer. Through *MET* amplification, the poorly differentiated early cancer progressed to advance cancer and metastasis [26].

In this study, we developed a convolution neural network (CNN) model, DeepIC50, to predict drug responsiveness by addressing not only three-class problem but also high dimensional features (~28,000). DeepIC50 is a 1-dimensional CNN (1D CNN) model that took 1-dimensional vectors as input. In DeepIC50, we integrated both genomics profiles and drug molecular features (including fingerprints) from massive drug treatments data on cancer cell lines. Our model showed better accuracy than the other baseline models including “2-dimensional convolution neural network” (2D CNN), support vector machine (SVM), ridge classifiers, and extreme gradient boosting (XGBoost). We also applied DeepIC50 and SVM to a GC patient dataset for further validation.

## 2. Results

### 2.1. Overview

**Figure 1:**
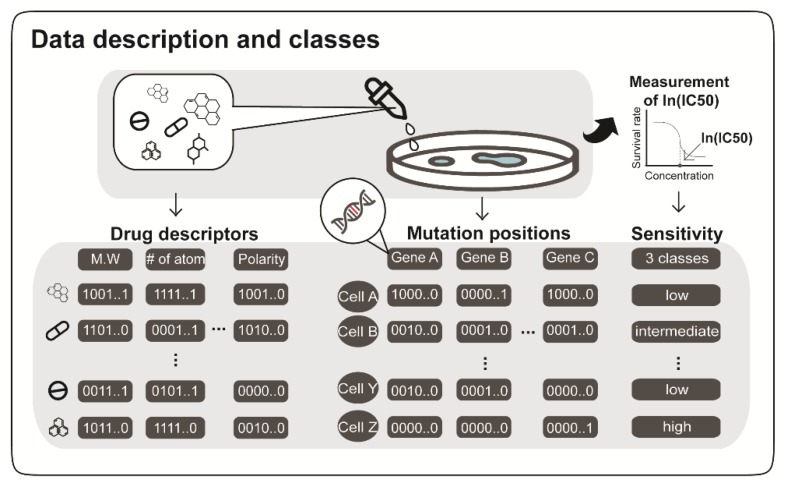
Overview of input data. DeepIC50 (equivalently, 1D convolution neural network (CNN)) and four baseline models predicted logarithms of half-maximal drug concentrations, ln(IC50)s, using genomics profiles of cancer cell lines and drugs’ molecular fingerprints as inputs. Drug molecular descriptors, including molecular weights, polarity, and molecular fingerprints were calculated by PaDEL [29]. Genomics profiles were represented by mutation statuses (presence or absence) in genomic positions of protein-coding genes. The ln(IC50) values of drugs used to treat cancer cell lines were grouped into three classes (see column sensitivity): high responsiveness (class 0), intermediate responsiveness (class 1), and low responsiveness (class 2).

**Figure 2:**
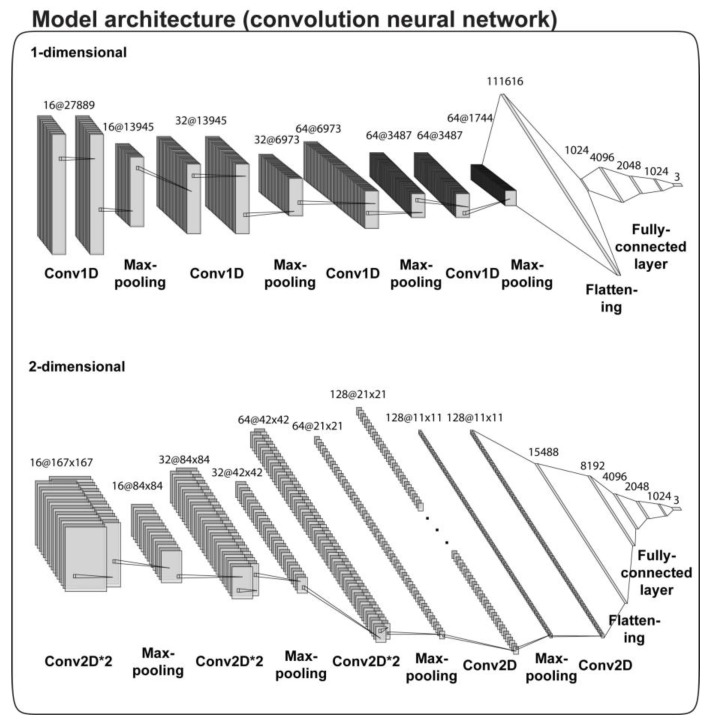
Architecture of DeepIC50 and 2D CNN. It represented our network structure (refers to 4.2 in materials and methods section in detail).

A drug’s half maximal inhibitory concentration (IC50) of cancer cell viability is widely used as a measure of its potency. Thus, higher drug potency results in lower IC50 values, in terms of cell viability. In this study, IC50 values of cancer cell viability were categorized to three classes: high (class 0; low IC50s), intermediate (class 1; intermediate IC50 values), and low (class 2; high IC50s) responsiveness (or efficacy). For prediction, input data consisted of mutation statuses of cancer cells, and chemical properties of compounds, leading to input vectors of the models (Figure 1). For this, we split the Genomics of Drug Sensitivity in Cancer (GDSC) dataset [10] into training and test sets. For another independent model evaluation, the Cancer Cell Line Encyclopedia (CCLE) [12] was used. Moreover, we inspected whether high potency drugs for GC cell lines aligned to similar predicted responses for a GC patient dataset in The Cancer Genome Atlas (TCGA) [28]. For this purpose, we used 2D CNN, SVM, ridge, and XGBoost, as baseline models. The structure of the CNN models (DeepIC50, 2D CNN) is depicted in Figure 2.

### 2.2. Model Comparisons of DeepIC50 against Other Baselines, in the GDSC Dataset

**Figure 3:**
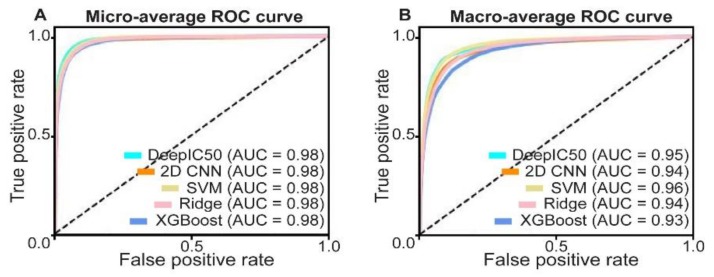
Performance of the five methods (DeepIC50, 2D CNN, support vector machine (SVM), ridge classifier, and XGBoost) in the Genomics of Drug Sensitivity in Cancer (GDSC) test set. Micro-average area under the curves (AUCs) (**A**) and macro-average AUCs (**B**) were represented by receiver operating characteristics (ROC) curves.

We next trained DeepIC50 and the other baseline models, based on the GDSC training set. In the GDSC test set, area under the curves (AUCs), for all the models, were determined. Since our problem is a three-class prediction, AUC of binary class prediction cannot be considered. So, micro- and macro-averages [30] were used to obtain receiver operating characteristics (ROC) curves (Figure 3). For the GDSC test set, the AUCs of micro- and macro-averages of the DeepIC50 were 0.98 and 0.95, respectively, 2D CNN were 0.98 and 0.94 respectively, and the micro- and macro-averages for SVM were 0.98 and 0.96, respectively. For the ridge classifier, micro- and macro-averages were 0.98 and 0.94 respectively, while for XGBoost, the micro- and macro-averages were 0.98 and 0.93, respectively (Figure 3A,B). In confusion matrix, DeepIC50 was better in comparison to the other baseline models (Supplementary Tables S1, S2, S3, S4, and S5). SVM showed slightly higher macro-average AUCs, in comparison to the baseline models.

The AUCs of one-vs-rest decisions (henceforth, OVRs), i.e., one class vs. the others, were obtained in the test sets (Supplementary Figure S1). The AUCs of OVRs, for DeepIC50 from 0.94 to 0.97; those of 2D CNN, ranged from 0.92 to 0.96; those of SVM, ranged from 0.94 to 0.97; those in ridge classifier from 0.91 to 0.97; and those in XGBoost from 0.90 to 0.96.

### 2.3. Model Comparison in an Independent Validation Dataset, CCLE

**Figure 4:**
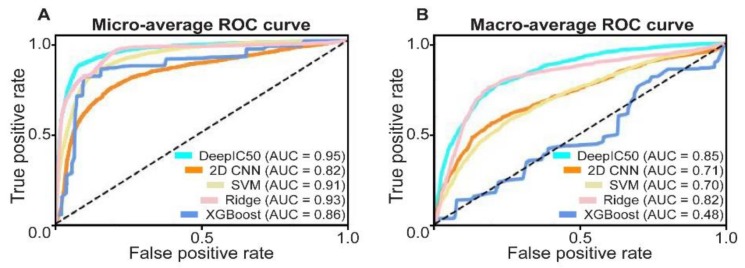
Performance of DeepIC50, 2D CNN, SVM, ridge classifier, and XGBoost in the Cancer Cell Line Encyclopedia (CCLE) dataset. Micro-average (**A**) and macro-average (**B**) AUCs were, along with ROC curves, represented for the five models.

By using the five models trained by the GDSC dataset, we used the whole CCLE [12] dataset for an independent validation. We applied the five models from the GDSC training set, to the CCLE dataset. In the CCLE dataset, the AUCs of micro-averages of DeepIC50, 2D CNN, SVM, ridge classifier, and XGBoost were 0.95, 0.82, 0.91, 0.93, and 0.86, respectively (Figure 4A), while the AUCs of macro-averages were 0.85, 0.71, 0.70, 0.82, and 0.48, respectively (Figure 4B). However, the confusion matrices of all methods (Supplementary Tables S6, S7, S8, S9, and S10), XGBoost did not predict any cases of class 0. Of the AUCs of OVRs, DeepIC50 ranged from 0.82 to 0.87, while the other baseline models ranged from 0.43 to 0.83 (Supplementary Figure S2). Overall, considering both performance comparison results in the GDSC test set and the CCLE dataset (Figure 3, Figure 4), DeepIC50 showed consistently good performances without AUC variability over the other baseline models. It is noted that the CCLE dataset did not contain class 2 cases.

### 2.4. Application of DeepIC50 and SVM to a TCGA GC Patient Dataset

We next assessed whether responsiveness of GC cell lines treated with potent drugs were comparable to prediction in GC patients in The Cancer Genome Atlas (TCGA) [28].

**Figure 5:**
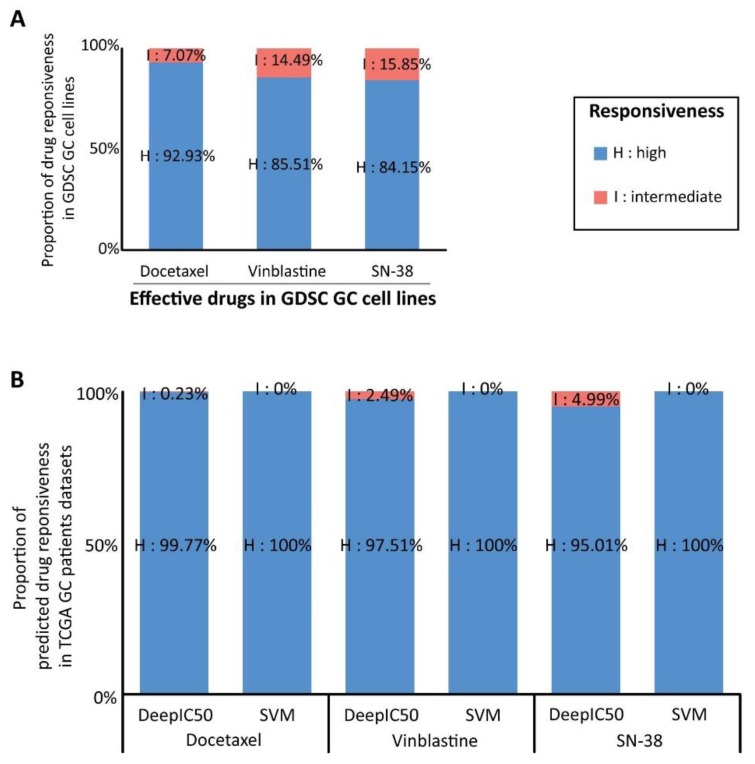
DeepIC50 and SVM application to a The Cancer Genome Atlas (TCGA) gastric cancer (GC) patient dataset (*n* = 441), to inspect whether drug effectiveness observed in GC cell lines was aligned to predicted responsiveness, by DeepIC50, for TCGA GC patients. (**A**) Distribution of the observed responsiveness for the three selected potent drugs in GDSC GC cell lines. We selected three effective drugs (refer to method section) in the GDSC GC cell line ln(IC50) experiments. The drugs showed “high responsiveness” in the most of the cell lines, and “intermediate responsiveness” in the minority of the cell lines. (**B**) Distribution of predicted drug responsiveness in TCGA GC patients. Prediction of patient drug responsiveness by DeepIC50 was more similar to drug responsiveness pattern in GC cell lines, in comparison to the SVM model. DeepIC50 predicted minor population of the patients to be “intermediate responsiveness”, but SVM predicted all the patients to be “high responsiveness” class without any other classes. Considering a single drug cannot fit all patients due to GC heterogeneity, DeepIC50 prediction was assumed to be reasonable.

Looking into the GC cell lines and their drugs in the GDSC dataset, three potent drugs (docetaxel, vinblastine, and SN-38) were selected (Figure 5A). These drugs indicated “high responsiveness” in the majority of the GC cell lines in the GDSC dataset. These drugs also showed “intermediate responsiveness” in the minority of the cell lines (Figure 5A). It was expected that these potent drugs showed similarity in the GC patients: the majority of the patients were predicted to “high responsiveness”, but the minority of the patients should be predicted to the other responsiveness classes (intermediate and low responsiveness). The expectation is reasonable, considering heterogeneity of GC patients and cell lines [28,31] confers the different responsiveness. The expectation was observed in DeepIC50 responsiveness predictions on the GC patients (Figure 5B). However, SVM did not show the pattern like DeepIC50, and predicted all the patients to “high responsiveness” without exception. Thus, DeepIC50 over SVM was more realistic in patient drug response prediction. In another CNN model, 2D CNN, the expectation was observed, while the expectation not observed in the other traditional models (ridge, XGBoost; Supplementary Figure S3).

## 3. Discussion

In this study, we developed a 1D CNN model, DeepIC50, for predicting three classes of drug potency, by integrating human cancer cell line genomics information (mutations) and drugs’ molecular descriptors. As compared to four baseline models (2D CNN, SVM, ridge classifier, and XGBoost), DeepIC50 demonstrated robustness and performance for multiple-class responsiveness prediction for multiple test sets including the TCGA GC patient dataset.

Here, we describe how DeepIC50 can be applied in two modes [1,2,3]. First, it is not realistic to measure drug responsiveness by treating patients; instead, cell line experiments, as proxies for cancer patients, would be highly advantageous [9]. Thus, this would be a more realistic strategy to construct drug responsiveness models using cancer cell lines, and then applying those models to a concatenation vector of a patient mutation status vector and a drug chemical property vector. These models could subsequently predict patient responsiveness to a drug. We applied this strategy to align drug response of cell lines to patient response prediction (see previous section entitled “Application of DeepIC50 and SVM to the TCGA GC patient dataset”).

Second, to predict the potency of a new drug, to a new cell line, we cannot utilize separate models for individual drugs when the separate models for either the new drug or the new cell line are unavailable. In the case, one could construct one-model for all drugs like our DeepIC50 model [3]. For example, concatenating mutation statuses of the new cell line and chemical properties of the new drug results in a input vector, which feeds into one-model to DeepIC50. Then, it predicts responsiveness for the potency of the new drug, to the new cell line.

For the implementation of drug responsiveness prediction, high dimensionality of cell line genomic profiles has been challenging [9], making deep learning a superior approach [17]. Likewise, this study showed that CNN good ways for multi-class prediction, by incorporating not only genomic profiles (mutations), but also drug chemical properties. In the future, machine learning should incorporate diverse genomics events, including copy number alterations, in addition to gene expression profiles of cell lines, as well as cell line clinical information for better predicting drug responsiveness.

In this study, we developed a 1D CNN model, DeepIC50, for predicting drug responsiveness, based on genomics (e.g., mutation statuses) and drug chemical properties, resulting in better performance, in comparison to the other baseline models. Such approaches may have application to this new age of “precision medicine.”

## 4. Materials and Methods

### 4.1. Dataset for Training and Test Sets

Cell line genomics information, drug molecular features/fingerprints, and logarithm of IC50 (henceforth, ln(IC50)) were from Genomics in Drug Sensitivity in Cancer (GDSC, https://www.cancerrxgene.org) [11], and cell line mutation profiles (CMPs) were from the COSMIC Cell Line Project (CCLP, https://cancer.sanganger.ac.uk). CMPs indicated the mutational statuses (e.g., presence or absence) of 21,213 genomic positions. Molecular descriptors of a drug included 6543 features (including the number of atoms, molecular weights, polarity, geometric features, electrostatic features, and 1-, 2-, and 3-dimensional molecular fingerprints). Drug molecular descriptors were calculated by PaDEL [29], taking drugs’ molecular file formats as input.

For drug features, we dropped the decimal point and then converted the values to binary, using R version 3.5.2 and Rstudio version 1.1.442. In the GDSC, there were 194,750 cell line-drug ln(IC50) pairs available. For each pair, we concatenated the CMPs and drug information features into a one-dimensional vector, removing redundancy and filtering out data having missing information. The resultant data amounted to 160,375 input vectors. ln(IC50) values of the 160,375 cases were labeled as three classes, by dividing the ln(IC50) ranges evenly. The ln(IC50) less than 2.36 were assigned to class 0 (high responsiveness); ln(IC50) between 2.36 and 5.26 to class 1 (intermediate responsiveness); and ln(IC50) greater than 5.26 to class 2 (low responsiveness). Then, the 160,375 were divided to 80% and 20% as the training set (128,300 cases) and test set (32,075 cases), respectively. In the test set, there were 2954 cases, 26,608 cases, and 2513 cases for classes 0, 1, and 2, respectively.

As another independent validation set, we processed the Cancer Cell Line Encyclopedia (CCLE) dataset [12] in the same way as the GDSC dataset. The CCLE also had drug names, cell line names, and ln(IC50) values. The CCLE dataset was entirely used as a validation set for the DeepIC50 model trained from the GDSC training set. We calculated drug molecular descriptors by using chemical structures (in PubChem [32]), corresponding to the drug name. Mutation statuses of the cell lines were assumed to be same as those of the cell lines in the GDSC genomics data. After redundancy removal and filtering, 153 cancer cell lines and 24 drugs were available, resulting in 2814 cases (i.e., one-dimensional vectors) consisting of cell line mutation statuses, drug molecular descriptors, and drug responsiveness classes. Subsequently, there were 544, 2270, and 0 cases for classes 0, 1, and 2, respectively.

### 4.2. DeepIC50 Construction

Referring to a binary drug responsiveness classification CNN, CDRScan [17], we extended to a 1D CNN with 1-dimensional input vectors for predicting multi-class drug responsiveness CNN.

The DeepIC50 model had six convolution layers, four max-pooling layers, and five fully connected layers, in its network structures (Figure 2). In each convolution layer, the filter size was 11, with stride 1. The number of filters is 16, 32, and 64 for every two convolutional layers. The activation function was Relu, and the max-pooling was size 2, with stride 2. The fully connected layers consisted of 1024, 2048, 4096, 2048, and 1024 nodes (Figure 2, Supplementary Table S11). The CNN was implemented by Keras and trained by a batch size of 25 and 100 epochs. Loss function was categorical_crossentropy of the Keras, the optimizer was keras.optimizers.Adam of Keras and metrics was accuracy. DeepIC50 was trained from the GDSC training set, and then tested using not only the GDSC test set but also the entire CCLE dataset. DeepIC50 code is available at https://github.com/labnams/DeepIC50.

### 4.3. 2-Dimentional Convolution Neural Network (2D CNN) Baseline Model

The input data for the multi-class 2D CNN model was then transformed from the concatenated one-dimensional vector to a 2-dimensional matrix, 167 by 167.

The 2D CNN had eight convolution layers, four max-pooling layers, and four fully connected layers, in its network structures (Figure 2). In each convolution layer, the filter size was 11 by 11, with stride 1. The number of filters started at 16 and was doubled every time the max pooling passed except last max pooling. The activation function was Relu, and the max-pooling was size 2 × 2, with stride 2. The fully connected layers consisted of 8192, 4096, 2048, and 1024 nodes (Figure 2, Supplementary Table S12). The CNN was implemented by TensorFlow (v1.12.0) and trained by a batch size of 125 and 100 epochs. Cost function was softmax_cross_entropy_with_logits of the TensorFlow and the optimizer was tf.train.AdamOptimizer of TensorFlow. 2D CNN also trained and tested the same way as DeepIC50.

### 4.4. Other Baseline Models: SVM, Ridge Classifier, and XGBoost

For SVM construction, the “linearSVC” function in the scikit-learn package (v0.20.2) was used, and probabilities for the three classes were calculated by the “calibratedclassifierCV” function in the package. The “linearSVC” function took the concatenated one-dimensional vectors as input, to predict the three-class drug responsiveness. In “linearSVC” function, “dual” was set to false, and all the other parameters were set to default options. The “cv” of “calibratedclassifierCV” function was set to five [17]. To implement XGBoost, the python XGBoost package (v0.81) was used with default options, excepting setting “objective” to “multi:softprob” for multi-class classification. The ridge classifier was implemented by the scikit-learn linear model package with default options except setting “alpha”, “copy_X”, and “solver” to 0.0001, false, and lsqr (least-squares routine) respectively. XGBoost and ridge classifier took the concatenated one-dimensional vectors as input, predicting the three classes of drug responsiveness.

### 4.5. Performance Comparisons of the Five Models

After training the five models from the GDSC training set, we used the GDSC test set and an independent validation set, from the CCLE (https://portals.broadinstitute.org/ccle). Performance comparisons were measured by micro-average and macro-average-AUCs [33]. We used scikit-learn package. In addition, for each class, an AUC for one-vs-rest (OVR; e.g., a class vs. the other classes) was calculated.

In an ROC curve, *x*-axis was false positive rates (FPRs), and *y*-axis true positive rates (TPRs). Due to three-class problem, three OVR classifications were available to produce three FPRs and three TPRs. Given a TPR in *x*-axis in the ROC curve, average of the three FPRs for the three OVR classifications corresponding to the TPR were calculated, and then the pair of the TPR and the average was plotted. The plot was macro-average ROC, and the AUC of the plot was a macro-average AUC.

In a set, an actual responsiveness class of *k*-th case set to one-hot encoded vector ***y****_k_* (*k* = 1, .., *n*; *n*: the number of total cases). When the actual class of the *k*-th case was 0, ***y****_k_* was encoded to (1,0,0); when 1, ***y****_k_* to (0,1,0); and when 2, ***y****_k_* to (0,0,1). The score vector for the three classes predicted by a model for the *k*-th case set to ***s***_k_. The ***s***_k_ consisted of three elements: the first element was the predicted score of the case being assigned to class 0; the second element the predicted score of the case being assigned to class 1; and the third element the predicted score of the case being assigned to class 2. The operator “||” was defined as concatenation of its adjacent two vectors. We defined two concatenated vectors, ***y*** and ***s***. The ***y*** set to ***y***_1_|| ***y***_2_ || .. || ***y**_k_* || .. || ***y**_n_*. The ***s*** set to ***s***_1_|| ***s***_2_ || .. || ***s**_k_* || .. || ***s**_n_*. The scikit-learn python package used the actual one-hot encoded vector concatenation ***y*** and the predicted score vector concatenation ***s***, and subsequently plotted a micro-average ROC curve with micro-average AUC.

### 4.6. Selection of Potent Drugs Observed in GC Cell Lines

To find potent drugs in GC cell lines, we looked into responsiveness classes of drugs in the GC cell lines from the GDSC dataset. For each drug, counting the cell lines having class 0 (high) responsiveness, in the GC cell lines, we obtained the top three drugs in descending order of the counts, regarded as potent drugs in GC cell lines.

### 4.7. Drug Responsiveness Prediction in the TCGA GC Patients

We obtained 441 GC patients from the GDC Legacy Archive (https://portal.gdc.cancer.gov/legacy-archive), for processing, in addition to the data for patient mutation statuses. We concatenated the mutation statuses of a patient and the molecular descriptors of a drug, resulting in a one-dimensional input vector. This input vector was fed into models to predict drug responsiveness class. It was noted that the models were pre-trained in the training set in the GDSC.

## 5. Conclusions

We constructed cell viability IC50 prediction models based on pan-cancer cell lines, not a specific cancer type. In the line, this model could be applicable to other cancer type patients. However, in this study, we selected GC patients as an example for application, but further application in other cancer type patients is awaited, which is our limitation in this study. Our model, DeepIC50, also has another limitation. Drug potency should consider diverse pharmacological parameters including Lipinski’s rule of five [34,35,36]. “Absorption, Distribution, Metabolism, Excretion, and Toxicity” (ADME-Tox) related parameters including drug (or functional chemical group) effects on certain tissues/organs should be considered [34,35,36]. These diverse parameters should be considered in further development of deep learning based cell viability IC50 prediction.

## Supplementary Materials

Supplementary Materials can be found at https://www.mdpi.com/1422-0067/20/24/6276/s1.
