# Supplementary material for: A Deep Learning Model for Cell Growth Inhibition IC50 Prediction and Its Application for Gastric Cancer Patients
Source: Int J Mol Sci. 2019 Dec 12;20(24):6276. doi: 10.3390/ijms20246276 (PMC6941066; doi:10.3390/ijms20246276)
Supplement: Supplementary file 1 [file ijms-20-06276-s001.pdf]

A deep learning model for cell growth inhibition IC50 prediction and its application for gastric cancer patients

Joo *et al*.

Supplementary Information

Supplementary Figures S1 through S3

Supplementary Tables S1 through S12

Supplementary Figure S1. Performance of the all methods in the GDSC test set. AUC of the class versus other classes (i.e., one-vs-rest) was obtained.

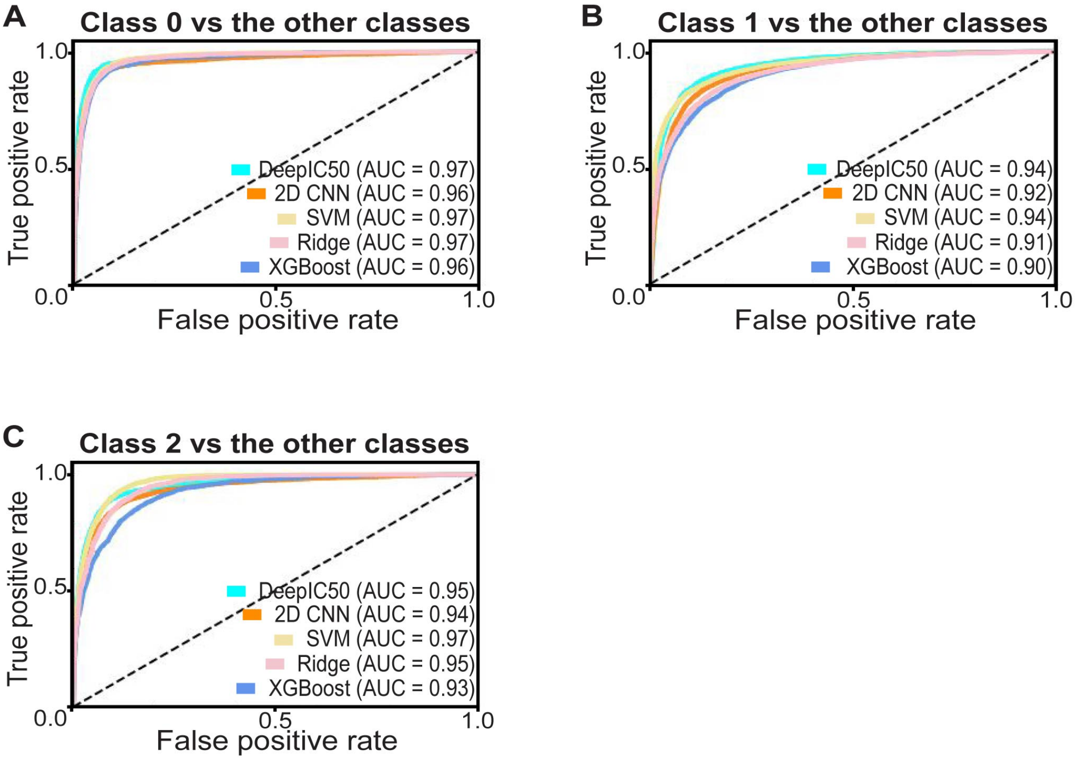

Supplementary Figure S2. Performance of the all methods in the CCLE set. AUC of the class versus other classes (i.e., one-vs-rest) was obtained. It is noted that the CCLE dataset did not have class 2 cases.

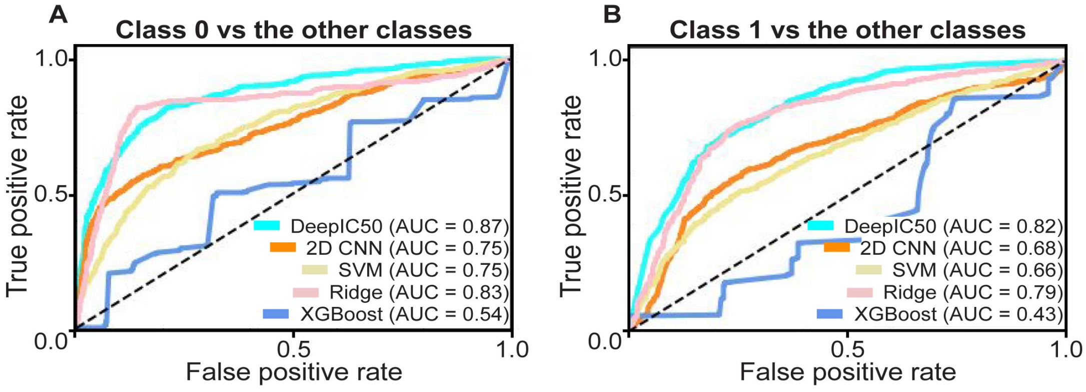

Supplementary Figure S3. Distribution of patient drug responsiveness predicted by all the methods in the TCGA GC patient dataset.

A

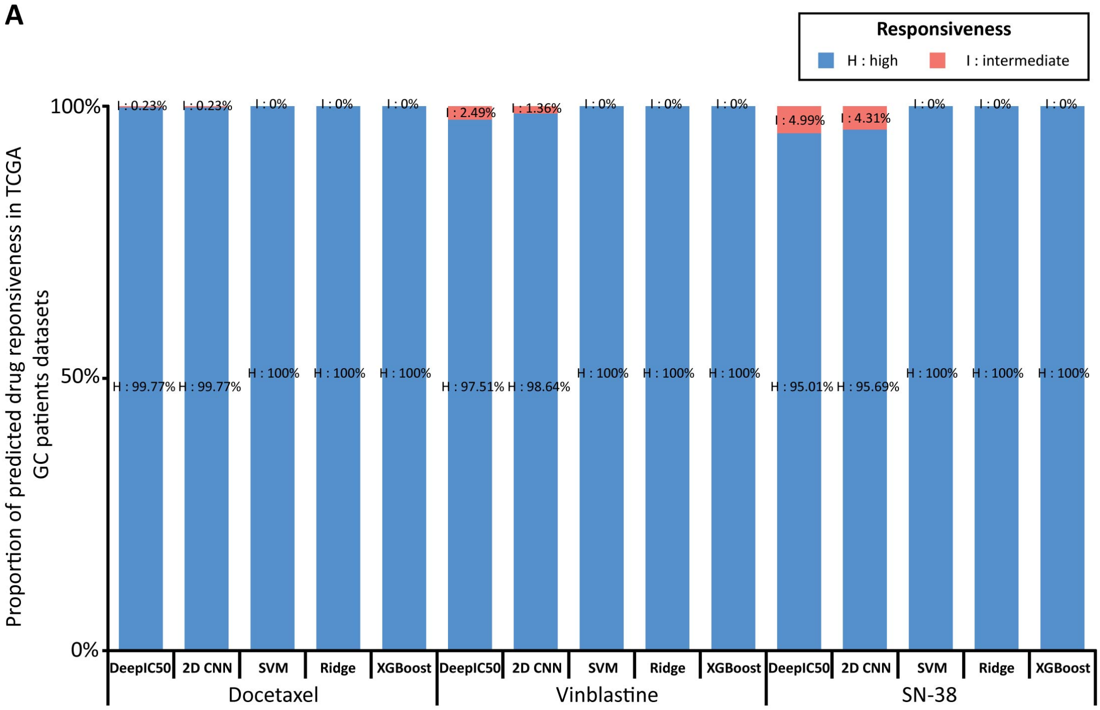

Supplementary Table S1. Confusion matrix of DeepIC50 in the GDSC test set.

Observed \ Predicted	Class 0	Class 1	Class 2
Class 0	2,123	831	0
Class 1	460	25,644	504
Class 2	0	1,077	1,436

Supplementary Table S2. Confusion matrix of 2D CNN in the GDSC test set.

Predicted / Observed	Class 0	Class 1	Class 2
Class 0	2,171	783	0
Class 1	677	25,170	761
Class 2	1	1,025	1,487

Supplementary Table S3. Confusion matrix of SVM in the GDSC test set.

Observed \ Predicted	Class 0	Class 1	Class 2
Class 0	2,103	851	0
Class 1	639	25,682	287
Class 2	0	1,408	1,105

Supplementary Table S4. Confusion matrix of ridge classifier in the GDSC test set.

Predicted / Observed	Class 0	Class 1	Class 2
Class 0	2,118	836	0
Class 1	795	25,478	335
Class 2	0	1,568	945

Supplementary Table S5. Confusion matrix of XGBoost in the GDSC test set.

Predicted / Observed	Class 0	Class 1	Class 2
Class 0	1,976	978	0
Class 1	663	25,698	247
Class 2	0	1,683	830

Supplementary Table S6. Confusion matrix of DeepIC50 in the CCLE test set.

Observed \ Predicted	Class 0	Class 1	Class 2
Class 0	210	333	1
Class 1	52	2,192	26
Class 2	0	0	0

Supplementary Table S7. Confusion matrix of 2D CNN in the CCLE test set.

Observed \ Predicted	Class 0	Class 1	Class 2
Class 0	159	325	60
Class 1	49	1,781	440
Class 2	0	0	0

Supplementary Table S8. Confusion matrix of SVM in the CCLE dataset.

Observed \ Predicted	Class 0	Class 1	Class 2
Class 0	23	508	13
Class 1	8	2,186	76
Class 2	0	0	0

Supplementary Table S9. Confusion matrix of ridge classifier in the CCLE data set.

Predicted / Observed	Class 0	Class 1	Class 2
Class 0	1	541	2
Class 1	0	2,262	8
Class 2	0	0	0

Supplementary Table S10. Confusion matrix of XGBoost in the CCLE data set.

Observed \ Predicted	Class 0	Class 1	Class 2
Class 0	0	544	0
Class 1	0	2270	0
Class 2	0	0	0

Supplementary Table S11. The number of parameters in the DeepIC50 model.

Layer (type)	Output Shape	Parameters
Input Layer	(None, 27889, 1)	0
Convolution Layer 1	(None, 27889, 16)	192
Batch normalization	(None, 27889, 16)	64
Convolution Layer 2	(None, 27889, 16)	2,832
Batch normalization	(None, 27889, 16)	64
Max Pooling 1	(None, 13945, 16)	0
Convolution Layer 3	(None, 13945, 32)	5,664
Batch normalization	(None, 13945, 32)	128
Convolution Layer 4	(None, 13945, 32)	11,296
Max Pooling 2	(None, 6973, 32)	0
Batch normalization	(None, 6973, 32)	128
Convolution Layer 5	(None, 6973, 64)	22,592
Batch normalization	(None, 6973, 64)	256
Max Pooling 3	(None, 3487, 64)	0
Convolution Layer 6	(None, 3487, 64)	45,120
Batch normalization	(None, 3487, 64)	256
Max Pooling 4	(None, 1744, 64)	0
Flatten	(None, 111616)	0
Fully Connected Layer 1	(None, 1024)	114,295,808
Batch normalization	(None, 1024)	4,096
Dropout 1	(None, 1024)	0
Fully Connected Layer 2	(None, 2048)	2,099,200
Batch normalization	(None, 2048)	8,192
Dropout 2	(None, 2048)	
Fully Connected Layer 3	(None, 4096)	8,392,704
Batch normalization	(None, 4096)	16,384
Dropout 3	(None, 4096)	0
Fully Connected Layer 4	(None, 2048)	8,390,656
Batch normalization	(None, 2048)	8,192
Dropout 4	(None, 2048)	0
Fully Connected Layer 5	(None, 1024)	2,098,176
Batch normalization	(None, 1024)	4,096
Dropout 5	(None, 1024)	0
Output Layer	(None, 3)	3,075
Total parameters		135,409,171

Supplementary Table S12. The number of parameters in the 2D CNN model.

Layer (type)	Output Shape	Parameters
Input Layer	(None, 167, 167, 1)	0
Convolution Layer 1	(None, 167, 167, 16)	1,952
Batch normalization	(None, 167, 167, 16)	64
Convolution Layer 2	(None, 167, 167, 16)	30,992
Batch normalization	(None, 167, 167, 16)	64
Max Pooling 1	(None, 84, 84, 16)	0
Convolution Layer 3	(None, 84, 84, 32)	61,984
Batch normalization	(None, 84, 84, 32)	128
Convolution Layer 4	(None, 84, 84, 32)	123,936
Batch normalization	(None, 84, 84, 32)	128
Max Pooling 2	(None, 42, 42, 32)	0
Convolution Layer 5	(None, 42, 42, 64)	247,872
Batch normalization	(None, 42, 42, 64)	256
Convolution Layer 6	(None, 42, 42, 64)	495,680
Batch normalization	(None, 42, 42, 64)	256
Max Pooling 3	(None, 21, 21, 64)	0
Convolution Layer 7	(None, 21, 21, 128)	991,360
Batch normalization	(None, 21, 21, 128)	512
Max Pooling 4	(None, 11, 11, 128)	0
Convolution Layer 8	(None, 11, 11, 128)	1,982,592
Batch normalization	(None, 11, 11, 128)	512
Flatten	(None, 15488)	0
Fully Connected Layer 1	(None, 8192)	126,885,888
Batch normalization	(None, 8192)	32,768
Fully Connected Layer 2	(None, 4096)	33,558,528
Batch normalization	(None, 4096)	16,384
Fully Connected Layer 3	(None, 2048)	8,390,656
Batch normalization	(None, 2048)	8,192
Fully Connected Layer 4	(None, 1024)	2,098,176
Batch normalization	(None, 1024)	4,096
Output Layer	(None, 3)	3,075
Total parameters		174,936,051
